# Understanding primary care-oncology relationships within a changing healthcare environment

**DOI:** 10.1186/s12875-019-1056-y

**Published:** 2019-11-28

**Authors:** Jennifer Tsui, Jenna Howard, Denalee O’Malley, William L. Miller, Shawna V. Hudson, Ellen B. Rubinstein, Jeanne M. Ferrante, Alicja Bator, Benjamin F. Crabtree

**Affiliations:** 1 Division of Population Science, Rutgers Cancer Institute of New Jersey, Rutgers, the State University of New Jersey, 195 Little Albany, New Brunswick, NJ 08903 USA; 2 Department of Family Medicine and Community Health, Rutgers Robert Wood Johnson Medical School, Rutgers, the State University of New Jersey, 112 Paterson St, New Brunswick, NJ 08901 USA; 3 Lehigh Valley Health Network, University of South Florida Morsani College of Medicine, 1247 S. Cedar Crest Blvd., Allentown, PA 18103 USA; 4 Department of Sociology and Anthropology, North Dakota State University, 428 Minard Hall, 1210 Albrecht Boulevard, Fargo, ND USA

**Keywords:** Cancer care/oncology, Primary care, Chronic disease management, Healthcare delivery, Physician relationships

## Abstract

**Background:**

Management of care transitions from primary care into and out of oncology is critical for optimal care of cancer patients and cancer survivors. There is limited understanding of existing primary care-oncology relationships within the context of the changing health care environment.

**Methods:**

Through a comparative case study of 14 innovative primary care practices throughout the United States (U.S.), we examined relationships between primary care and oncology settings to identify attributes contributing to strengthened relationships in diverse settings. Field researchers observed practices for 10–12 days, recording fieldnotes and conducting interviews. We created a reduced dataset of all text related to primary care-oncology relationships, and collaboratively identified patterns to characterize these relationships through an inductive “immersion/crystallization” analysis process.

**Results:**

Nine of the 14 practices discussed having either formal or informal primary care-oncology relationships. Nearly all formal primary care-oncology relationships were embedded within healthcare systems. The majority of private, independent practices had more informal relationships between individual primary care physicians and specific oncologists. Practices with formal relationships noted health system infrastructure that facilitates transfer of patient information and timely referrals. Practices with informal relationships described shared commitment, trust, and rapport with specific oncologists. Regardless of relationship type, challenges reported by primary care settings included lack of clarity about roles and responsibilities during cancer treatment and beyond.

**Conclusions:**

With the rapid transformation of U.S. healthcare towards system ownership of primary care practices, efforts are needed to integrate strengths of informal primary care-oncology relationships in addition to formal system driven relationships.

## Background

It is estimated that 1.7 million new cancer cases were diagnosed in 2018, and the number of cancer survivors in the United States will reach over 18 million by 2020 [1, 2]. High quality care from cancer diagnosis through survivorship requires care delivery processes involving different sets of providers and care teams [3, 4]. Poor transitions from primary care to oncology following cancer diagnosis are associated with worse outcomes and lower survival [3, 5, 6]. Suboptimal care coordination – including information transfer and role negotiation among multiple clinicians – during survivorship also remains the norm [7, 8]. Shared-care or team-based care, where clinicians interact more interdependently and adaptively to achieve common goals, provide more comprehensive and guideline concordant-care for cancer survivors than when followed by oncology teams only [9, 10]. Yet, the mechanisms that impact relationship and team building between primary care and oncology settings are poorly understood and remain underexplored in health services research [6, 7, 11–14].

Over the last decade, there has been rapid health care delivery consolidation in the U.S., including the acquisition of independent physician practices into hospital and other integrated systems and payment models [15–17], which can have profound effects in primary care delivery, and management for cancer patients [18–20]. Similarly, shifts from independent oncology practices to larger system affiliations has led to new referral patterns and underlying infrastructure that can affect relationships between primary care and oncology settings [21, 22]. An understanding of existing primary care-oncology relationships within the context of dynamic health care reorganization, including both system-based and independently-owned primary care practices, is necessary to inform long-term strategies for managing the care of cancer patients and survivors.

In this analysis, we describe existing relationships between primary care and oncology settings and identify attributes contributing to strengthened primary care-oncology interfaces in diverse settings.

## Methods

### Study Sample & Design

This a secondary data analysis of qualitative data collected from 2015 to 2017 as part of a National Cancer Institute-funded mixed methods comparative case study of 14 innovative primary care practices throughout the U.S. The parent study was designed to examine attributes of these innovators and their strategies to deliver care for cancer survivors. Practice recruitment and data collection methods were described in detail in previous publications [23, 24]. Briefly, practices were selected from a national list of 151 workforce innovators compiled for the Robert Wood Johnson Foundation in 2011–2012. Most were National Committee for Quality Assurance (NCQA) level 3 Patient-Centered Medical Home practices, diverse in size, geographic location, and ownership. Trained researchers visited each practice for 10–12 business days and collected: (a) observational fieldnotes and key informant interviews on clinical workflow; (b) fieldnotes of “patient pathways” (shadowing cancer survivors through their visit); (c) 54 recorded and transcribed semi-structured interviews with clinicians and staff; and, (d) practice documents, such as standard operating procedures, patient educational materials and organizational mission statements. A Practice Information Form on practice characteristics (e.g., size, organization, patient population, services, payment structure, etc.) was completed by a practice leader. The Rutgers University Biomedical and Health Sciences Institutional Review Board approved the study and written informed consent was obtained from recorded interview participants.

### Research questions

We conducted a comparative case study analysis using data from the parent study to answer the following questions (1]: What are the key features of primary care and oncology relationships across health care settings, specifically regarding patients’ transitions into and out of acute cancer treatment? and (2) Which attributes of these relationships can be adapted to strengthen the quality of care transitions for cancer patients in other settings?

### Data analysis

The compiled dataset from the parent study, consisting of over 2000 pages of qualitative data, was managed and coded using Atlas.ti software (Version 7.5.3). Initially, we applied a data reduction strategy from the 2000 pages of qualitative data and extracted all text on the topic of relationships between primary care and oncology providers using the following key terms: “oncology”/“oncologist,” “cancer center,” “referral,” “cancer diagnosis,” and “cancer treatment.” Data were analyzed using a grounded theory inductive approach [25], which involves a series of iterative and interpretive immersion/crystallization cycles of reading and analyzing the data until patterns and themes emerge [26]. Two authors (JT and JH) independently read the reduced dataset to identify patterns. This second reading clarified which primary care clinicians at which practices spoke about their interactions with oncology. The two authors then wrote short case summaries that described each practice’s relationship with oncology and shared these with the larger research team, including the field researchers who collected the data, to reflect together on themes. We compared and contrasted each practice with our emergent themes and engaged in several iterative rounds of re-reading the data and collaboratively reflecting before agreeing on the final characterization of primary care-oncology relationships. Quantitative information on practice characteristics from the Practice Information Forms was examined and compiled into Table 1.

**Table 1:** Primary care practice charactristics by formal and informal primary care-oncology relationship

Practice ID	Location	Ownership	# of Primary Care Physicians	# of NPs or PAs	# of Patient Visits per Year	% Medicaid or Uninsured Patients	% Minority Patients
Formal Primary Care-Oncology Relationship							
P6	Rural	FQHC within Health System	8	8	33,233	19	4
P9	Urban	Capitated Non-Profit, Independent Practice	11	2	44,000	5	79
P10	Suburban	Academic- Hospital Health System	12	3	26,000	8	64
P11	Suburban	Veterans Administration	5	0	9151	0	unk
Informal Primary Care-Oncology Relationship							
P1	Suburban	Independent, Physician Owned	10	3	84,000	9	5
P2	Suburban	Independent, Physician Owned	6	2	19,933	10	10
P4	Rural	FQHC	7	2	25,000	unk	unk
P8	Suburban	Independent, Physician Owned	3	3	19,380	1	21
P14	Small city	Independent, Physician Owned	3	2	4771	60	7
No Relationship Mentioned							
P3	Suburban	Hospital Health System	7	4	37,828	8	6
P5	Suburban	Health System	3	1	9447	6	unk
P7	Urban	University Nurse-Led, FQHC	1	4	11,035	87	71
P12	Urban	Private, direct primary care	5	1	9557	32	unk
P13	Suburban	Private practice within network of independent practices	3	0	5791	0	unk

*FQHC* Federally Qualified Health Center, *unk* Unknown/Missing

## Results

The 14 practices were located in diverse settings (four urban, two in small cities, six suburban, two rural) across nine states (See Table 1). They represented various practice types, including five physician-owned (2 being Direct Primary Care models), three hospital or health system-owned, one Veteran’s Administration (VA) practice, one university nurse-led, one capitated non-profit, one practice within a network of independent primary care practices, and two Federally Qualified Health Centers (FQHCs). Other analyses of these 14 practices explored primary care physicians’ perspectives on cancer survivorship care [27] and barriers and opportunities for systematic cancer survivorship care in advanced primary care practices [24]. These prior analyses revealed a limited primary care identity within the broader healthcare context for cancer survivorship care and lack of ability of current information systems to support survivorship care. While these findings are relevant to the relationship between primary care and oncology, this analysis focuses explicitly, and with more depth, on understanding these practice’s specific relationships with oncologists.

Nine of the 14 practices (i.e. P1, P2, P4, P6, P8, P9, P10, P11, P14) discussed features of existing relationships with oncologists. Of these, four described formal relationships between their practice and oncologists or hospital oncology services (P6, P9, P10, P11). Formal relationships included either being within the same hospital-owned health system or having a financial contract with specific oncologists. Five practices, which were not based within hospital or health system-owned settings, spoke about relationships with oncology that relied more on personal relationships between individual physicians and oncologists (P1, P2, P4, P8, P14). We provide examples below to characterize the two types – formal versus informal relationships – and the impact of these relationships on cancer care transitions.

### Formal primary care-oncology relationships

The primary care practices with formal relationships with oncology settings, included an FQHC within a rural health system (P6), a hospital-owned academic residency (P10), a Veterans Administration (VA) community-based outpatient clinic (CBOC) (P11), and an independent, non-profit primary care practice that employed its own part-time specialists, including multiple oncologists (P9). Three case studies (two system-based; one independent), described below, characterize these formal primary care-oncology relationships.

### CASE #1: FQHC within rural system

P6 is a large FQHC within a 17-clinic health system that refers most cancer cases to the local hospital’s cancer center. The health system established a memorandum of understanding (MOU) between the primary care clinics and the hospital’s oncology department, with the purpose of improving communication around mutual patients’ care during cancer treatment and “to reduce duplicative services between primary and oncology care” after treatment, as described by one primary care physician. Another physician identified some of the problematic issues that had motivated creating a formalized process between these care settings:

Our MOU with oncologists really is trying to find the right relationship between primary care and the oncologists, and we are frustrated by two sets of things happening. One is the patients who really are failing on aggressive care continue to get it when they don’t need it and not being offered the palliative services. And then the other frustration is people 10 years out being called back every 4 months for more [tests] …

Reported benefits of having this formalized agreement included a reliable process of receiving oncology notes, a registry of shared patients, and consistent referral patterns, allowing for timely access to oncologists.

### CASE #2: hospital-owned academic residency clinic

Another example of a formal structured primary care-oncology relationship is P10, a large, urban hospital-owned academic residency clinic with an academic affiliation to a National Cancer Institute (NCI)-designated comprehensive cancer center, located a mile away. Nearly all oncology referrals are sent to the cancer center, which was spoken of highly by several physicians and staff.

Clinicians in P10 also mentioned that having a shared electronic health record (EHR) and email system has contributed to the positive relationship:

[I] think it’s a really great working relationship, but I mean I think the nice thing is that we’re connected by the Epic [EHR] system and email. We can see their visits. We can message them through the system. Patients get referred by the hospital or from here … I think it’s a really great relationship.

The referral of patients was in fact a reciprocal process between P10 and the cancer center. It was common for the cancer center to ask the practice to assign P10 clinicians for new patients seeking care at the center who did not currently have an affiliated primary care clinician.

### CASE #3: independent, non-profit clinic

While it was generally the case that structured primary care-oncology relationships occurred within system-based practices, P9 was the exception. P9 developed formal relationships with oncologists by hiring several onsite specialists, including two oncologists and a surgeon. Exchange of health information, electronically and verbally, was seamless both because primary care clinicians and in-house specialists used the same EHR and because they saw patients within the same clinic. The P9 surgeon explained: “[The typical] black box [between oncology and primary care] is better here because we can have ongoing communication.” A P9 oncologist highlighted that one of the advantages of having oncologists and primary care providers working together in the same practice is that patients get into treatment quickly after a diagnosis. One P9 oncologist was also employed by a large, academic hospital/cancer center, which added to the ease in communication between the primary care providers and external oncologists involved in patient care.

### Informal primary care-oncology relationships

There were five practices (P2, P4, P8, P1, P14) that did not have any kind of structured relationships with oncology, yet clinicians in these practices described having informal relationships with individual oncologists. All five were private, independent practices that had no health system-affiliation. The context that contributed to these clinicians’ relationships with oncologists varied across the five practices, but focused mainly on rapport-building and increased efforts for team-based approaches to care. Two case studies below characterize these informal primary care-oncology relationships:

### CASE #1: large, suburban, independent physician-owned practice

P1 is a large, privately-owned multi-specialty clinic with family- and internal medicine physicians as well as multiple specialists, most of whom sublease space from P1. Although it is located in close proximity to a cancer center, P1 physicians tended not to refer there. They felt the cancer center did not provide good care, so as one physician put it, she “voted with [her] feet long ago” and found, through trial and error, community oncologists who lived up to her expectations:

[T]hey’re communicating with me … they treat my patients with kindness, they’re happy to hear from me on the phone, and they’re sending me their thoughts … so it’s a whole team approach.

While P1 has the option of multiple oncology groups to refer to within the region, including the local cancer center, they chose to rely on oncologists with whom they felt they could have reciprocal communication and shared care of their patients.

### CASE #2: medium, suburban, independent physician-owned practice

P2 is a medium-sized, privately owned family medicine practice that has been in existence for 35 years. They have prided themselves in long-term patient relationships, often caring for multiple generations of entire families. Physicians at P2 have prioritized developing “good rapport” with specialists (including oncologists) and therefore decided to round at the hospital rather than using hospitalists. While most primary care physicians in the area have stopped rounding (i.e. when a physician sees patients in the hospital, sometimes with medical trainees, and discuss the overall course of treatment with other members of the health care team, including nurses and specialists) unless they have teaching obligations, P2 physicians chose to continue hospital rounding as a way to cultivate relationships with inpatient care providers. They were willing to invest in these relationships because, as the office manager explained, the P2 physicians “want to keep themselves in the loop [of their patients’ care].” Even though they are not owned by the hospital, physicians felt strongly that rounding was advantageous:

Being affiliated and knowing the doctors [at the hospital], being able to actually interact with them in front of the patients, knowing that you’re all involved in the care is great for them (patients).

Overall, despite their independence as a private practice, P2 developed ways to foster relationships and enhance communication with hospital oncologists.

## Discussion

This is one of few studies to examine characteristics of relationships between primary care and oncology settings within the recent healthcare environment. We found that primary care-oncology relationships situated within formal settings, indicated several common advantages, including having shared health information technology infrastructure, which allowed for rapid and secure patient information transfer between settings, as cited in prior studies [13, 28]. Additionally, shared information systems allowed primary care practices to stay connected to their patients during acute cancer treatment, and facilitated bidirectional referrals. Many characteristics of these relationships may be applicable and already in place between primary care and other specialists (e.g. cardiologists, endocrinologists) for co-managing conditions such as diabetes and hypertension. However, less is understood about the communication systems between primary care and oncology, particularly outside of vertically integrated health systems.

While formal primary care-oncology relationships benefited from the broader health system infrastructure, informal relationships had the advantage of increased rapport between providers in primary care and oncology settings. Although these personal primary care-oncology relationships were often tied to one individual within the organization and thus may not be sustainable long-term, they had the advantages of shared familiarity, trust, and commitment, characteristics that have been noted in other team-based care studies [29–31]. Given that rapid changes in primary care ownership across the U.S. are ongoing, it is important for health systems to encourage team-building between primary care and oncology settings, in addition to strengthening infrastructure for information transfer. Prior studies on relationships between providers, particularly within the context of telemedicine, have supported the need to establish interdependent relationships for optimal team-based care [32–35]. Insight from these case studies contributes to our growing understanding of features of both formal and informal relationships that are important for optimal management of a patient’s journey through the cancer care continuum.

Despite the advantages highlighted in either formal or informal relationships with oncologists, clinicians in all practices identified remaining care coordination challenges during acute and survivorship phases of cancer care [14]. For example, multiple primary care clinicians mentioned having limited participation in their patients’ care once they transitioned to oncology. There was a lack of clarity in the respective roles and responsibilities of primary care and oncology during care transition points. Lastly, several clinicians in both formal and informal settings mentioned a lack of guidance on appropriate follow-up care plans for survivors. National guidelines on cancer survivorship care indicate that long-term systematic care should take place within primary care settings as the number of cancer survivors continues to increase [36–39]. However, recent studies have shown implementation of systematic survivorship care is extremely limited within primary care practices [24, 40–42]. Substantial efforts to manage the care of cancer survivors systematically within primary care settings and to address barriers to this implementation are warranted [10, 43].

While this is one of few studies using empirical qualitative data to understand relationships between primary care and oncology, it is not without limitations. First, the focus on practice-level relationships rather than clinician relationships may have resulted in missing data on some individual primary care clinicians’ relationships with oncologists. This may explain why data from five practices did not reveal any stated primary care-oncology relationships. Furthermore, we did not collect data from oncology practices and were therefore unable to compare perspectives and institutional contexts between the two settings. Although best attempts were made to identify the most relevant individuals to interview regarding cancer care transitions, it is possible that some key informants were not available. Additionally, while our sample of innovative practices was diverse, it was not inclusive of all types of primary care settings. For example, we did not include a primary care practice that is part of a fully integrated health system, such as Kaiser Permanente. These systems may facilitate different relationships between primary and oncology care. Lastly, this study did not include measures of health care quality or outcomes at the patient-level and thus is limited in assessing how variations in primary care and oncology relationships impact clinical endpoints. These limitations notwithstanding, the current study provides important new data on interfaces and relationships between primary care and oncology that affects cancer care management.

## Conclusions

Improvements in the management of care transitions from primary care into and out of oncology are critical for achieving optimal care quality and outcomes for cancer patients and survivors. Advantages of strong relationships between generalists and specialists, in general, have been observed in studies as far back as Rhee and colleagues in 1980 [44] and as recently as National Cancer Institute–American Society of Clinical Oncology Teams in Cancer Care Project, specifically for team-based cancer care delivery [4]. While evidence is limited on the relative benefit of formal versus informal primary care-oncology relationships, the increasing number of system/hospital-owned practices may provide additional infrastructure to facilitate such relationships. Findings from this study provide evidence to monitor and further explore the impact of increasing integration of primary care and oncology practices with hospital settings and whether these trends improve communication strategies via more organized infrastructure over time. Furthermore, as the implementation of patient navigators continues to grow in diverse ways, including use of nurses, medical assistants, and community health workers [45–48], it is not yet clear whether any one of these strategies improve primary care and oncology relationships for the care of cancer survivors. As more practices are consolidated into health systems or bound to contractual relationships with specific oncology groups, fostering rapport and team-building among clinicians in these settings may be crucial for optimizing the potential of system-based supports [49, 50]. Increased research to understand health organizational differences that are associated with team rapport and quality and outcomes for cancer patients are needed [14, 51]. For example, building empirical evidence of the impact of fostering rapport and team building between primary care and oncologists in both formal and informal relationships will contribute to a much needed understanding of how care delivery teams can improve cancer outcomes [52]. Recent studies in building multidisciplinary care teams for cancer patients, through increased team meetings, have improved quality but not survival, suggesting more research is needed in this area [53]. In summary, these study findings provide support to build evidence for strategies to better integrate strengths of informal primary care-oncology relationships in addition to formal system driven relationships that are ongoing within the health care context.

## Data Availability

The dataset generated and analyzed during the current study is not publicly available because the detailed nature of this qualitative data could compromise participant anonymity.

## Abbreviations

CBOC: Community Based Outpatient Clinic
EHR: Electronic Health Record
FQHC: Federally Qualified Health Center
MOU: Memorandum of Understanding
NCQA: National Committee for Quality Assurance
VA: Veterans Administration
